# Emerging Targeted Therapy for Specific Genomic Abnormalities in Acute Myeloid Leukemia

**DOI:** 10.3390/ijms23042362

**Published:** 2022-02-21

**Authors:** Sung-Gi Chi, Yosuke Minami

**Affiliations:** Department of Hematology, National Cancer Center Hospital East, Kashiwa 2778577, Japan; schi@east.ncc.go.jp

**Keywords:** acute myeloid leukemia, *FLT3*, *IDH1*, *IDH2*, BCL-2, menin, *MLL* rearrangement, *NPM1*, *NUP98* fusion, SYK, *TP53*, CD47, *KIT*, *KRAS*, *NRAS*

## Abstract

We describe recent updates of existing molecular-targeting agents and emerging novel gene-specific strategies. FLT3 and IDH inhibitors are being tested in combination with conventional chemotherapy for both medically fit patients and patients who are ineligible for intensive therapy. FLT3 inhibitors combined with non-cytotoxic agents, such as BCL-2 inhibitors, have potential therapeutic applicability. The menin-MLL complex pathway is an emerging therapeutic target. The pathway accounts for the leukemogenesis in AML with *MLL*-rearrangement, *NPM1* mutation, and *NUP98* fusion genes. Potent menin-MLL inhibitors have demonstrated promising anti-leukemic effects in preclinical studies. The downstream signaling molecule SYK represents an additional target. However, the *TP53* mutation continues to remain a challenge. While the p53 stabilizer APR-246 in combination with azacitidine failed to show superiority compared to azacitidine monotherapy in a phase 3 trial, next-generation p53 stabilizers are now under development. Among a number of non-canonical approaches to *TP53*-mutated AML, the anti-CD47 antibody magrolimab in combination with azacitidine showed promising results in a phase 1b trial. Further, the efficacy was somewhat better in patients with the *TP53* mutation. Although clinical evidence has not been accumulated sufficiently, targeting activating *KIT* mutations and RAS pathway-related molecules can be a future therapeutic strategy.

## 1. Introduction

Acute myeloid leukemia (AML) is an aggressive hematologic malignancy often characterized by specific genomic alterations [1]. The standard treatment strategy for AML is largely consisted of intensive chemotherapy with or without hematopoietic stem cell transplantation. However, long-term survival can be achieved in only up to three-quarters of patients, even in the favorable risk group [2]. Molecular-targeted therapy has had a significant impact on clinical practice, especially for patients with specific genomic abnormalities. The fms-like tyrosine kinase 3 (FLT3) mutation, for example, is known as one of the major adverse prognostic factors in AML. Further, potent FLT3 inhibitors have improved clinical outcomes as a part of salvage/alternative therapy as well as in combination with intensive chemotherapy in patients with *FLT3*-mutated AML. For patients with iso-citrate dehydrogenase (IDH)-1 or -2 mutation, specific IDH inhibitors ivosidenib and enasidenib are generally well-tolerated and expects complete remission (CR) rates of 30–40% as monotherapy [3,4]. Recently, menin-related leukemogenesis especially in AML with mixed lineage leukemia 1 (*MLL*)-rearrangement have gathered attention and several preclinical studies have evaluated its specific inhibitors. In addition, anti-tumor protein p53 (TP53), KIT, and RAS strategies have developed both in hematologic malignancies and solid tumors. A number of genomic abnormalities have been identified as potential targets and some have shown promising data in preclinical/early-phase studies. Here, we discuss emerging novel therapeutic approaches for AML, especially those targeting specific genomic abnormalities.

## 2. Targeting Specific Mutant Genes

### 2.1. Fms-like Tyrosine Kinase 3 Mutation

FLT3 protein is a receptor tyrosine kinase expressed in normal hematopoietic progenitor cells. FLT3 is dimerized upon binding with FLT3 ligands (FLs) produced by bone marrow stromal cells, which results in phosphorylation of the tyrosine residues in the activation-loop (A-loop) then the downstream signaling follows [5]. The *FLT3* mutation is the most frequent genomic abnormality, accounting for approximately 30% of adult AML, and is associated with poor prognosis [1,6]. *FLT3* mutations are largely divided into two types: an internal-tandem duplication of the juxta-membrane domain-encoding region (ITD) and single nucleotide variants of the tyrosine kinase domain-encoding region (TKD). Generally, AML with *FLT3*-ITD has stronger proliferation advantages and better sensitivity to FLT3 inhibitors than those with *FLT3*-TKD. Although both *FLT3*-ITD and *FLT3*-TKD are activating mutations, *FLT3*-ITD consistently upregulates the Janus kinase (JAK)/signal transducer and activator of transcription (STAT) signaling and *FLT3*-TKD enhance the src homology region 2 domain-containing phosphatase 1 (SHP1) and SHP2 activity that negatively regulate JAK signaling [7,8], which partially explains why *FLT3*-ITD has showed more potent myeloproliferative advantages than the other [9,10].

Potent FLT3 inhibitors such as midostaurin (RATIFY trial [11]), gilteritinib (ADMIRAL trial [12]), and quizartinib (QuANTUM-R trial [13]) have demonstrated clinical benefits as a salvage monotherapy or in combination with conventional chemotherapy in phase 3 trials (Table 1). FLT3 inhibitors show an approximately 50% CR rates as well as prolongation of survival in patients with relapsed/refractory (R/R) AML. Although gilteritinib and quizartinib are currently indicated only for R/R cases, combination therapy with standard chemotherapy for patients with newly-diagnosed (ND) *FLT3*-mutant AML is now being evaluated in several phase 3 clinical trials (HOVON 156 AML/AMLSG 28-18 [14], NCT03836209 [15], QuANTUM-First [16]). The next-generation FLT3 inhibitor crenolanib is a promising novel agent [17] currently under evaluation in phase 3 randomized trials for patients with R/R [18] and ND *FLT3*-mutant AML [19].

FLT3 inhibitors may also have an important role in maintenance therapy after allogeneic hematopoietic transplantation (allo-HSCT), and several clinical studies have evaluated the efficacy of sorafenib or midostaurin. A systemic review of 7 such studies including six-hundred-eighty FLT3-mutated AML patients revealed that FLT3 inhibitor maintenance therapy significantly reduced the risk of post-transplant relapse by 65% (HR 0.35, 95% CI 0.23–0.51) and improved survival rates (HR 0.48, 95% CI 0.36–0.64) [20]. The result of a phase 3 trial evaluating the efficacy of gilteritinib as post-transplant maintenance therapy is anticipated [21].

Combinations with low-intensity chemotherapy have also been evaluated. Retrospective and early-phase studies have suggested the modest efficacy of FLT3 inhibitors plus azacitidine or low-dose cytarabine with response rates of one-fifth to one-quarter (Table 2) [22,23,24]. However, the randomized phase 3 LACEWING trial, which compared the combination of gilteritinib plus azacitidine with azacitidine monotherapy in patients with ND *FLT3*-mutated AML or myelodysplastic syndrome (MDS) who were ineligible for intensive chemotherapy, did not meet the primary endpoint (overall survival) [25].

A combination strategy with non-cytotoxic agents has also been developed. A patient-derived xenograft (PDX) mouse model experiment demonstrated that quizartinib in combination with the selective B-cell leukemia/lymphoma 2 (BCL-2) inhibitor venetoclax significantly prolonged survival of *FLT3*-mutant PDX mice compared with those receiving monotherapy [26]. This study also suggested that *FLT3*-ITD inhibition led to increased dependance on BCL-2 through indirect attenuation of B-cell lymphoma-extra large (BCL-XL) and myeloid-cell leukemia 1 (MCL-1) which are involved in anti-apoptotic mechanisms. Arsenic trioxide (ATO), one of the key drugs in treating acute promyelocytic leukemia and which induces differentiation of leukemic cells by enhancing *RARA* gene function, in combination with the FLT3 inhibitors showed a synergistic anti-leukemic effect in vivo along with reduced expression (or facilitated poly-ubiquitination) of FLT3 proteins [27]. A PDX model study suggested that inflammatory genes were upregulated in quizartinib-resistant FLT3-mutant leukemia, and co-administration of dexamethasone with quizartinib showed synergistic cell death in vivo [28].

### 2.2. Isocitrate Dehydrogenase Mutations

#### 2.2.1. Targeting *IDH1* and *IDH2* Mutations

Isocitrate dehydrogenase (IDH) 1 and IDH2 proteins are house-keeping enzymes involved in the tricarboxylic acid cycle in mitochondria. Mutant IDH1 and IDH2 confer an aberrant enzymatic activity, which converts alpha-ketoglutarate (alpha-KG) to the oncometabolite 2-hydroxyglutarate (2-HG) [29], resulting in epigenetic alterations that prevent hematopoietic differentiation [30,31]. *IDH* mutations impaired the histone demethylation that is required for cell differentiation through producing 2-HG [31]. Indeed, *IDH*1/2-mutated leukemic cells displayed global DNA hypermethylation and attenuated function of the alpha-KG-dependent enzyme Tet methylcytidine dioxygenase 2 (TET2) [30], which partially explains why IDH mutations and *TET2* mutation are mutually exclusive in AML.

Both *IDH1* and *IDH2* gene mutations are found in approximately 20% of AML [6] as well as 12% of myelodysplastic syndrome (MDS) especially in high-risk cases [32]. *IDH1*/*IDH2* mutations are also commonly found in high-grade gliomas and IDH1 inhibition prolonged disease control with favorable safety profile in a phase 1 clinical study [33]. The specific IDH1 inhibitor ivosidenib demonstrated a 21.6% CR rate in patients with R/R *IDH1*-mutated AML in a phase 1 study [34]. Ivosidenib also showed a 42.4% CR plus CR with partial hematologic recovery (CRh) rates as monotherapy [3] and a 60.9% CR rate in combination with azacitidine in patients with newly-diagnosed *IDH1*-mutated AML who were ineligible for standard therapy [35]. Similarly, the IDH2 inhibitor enasidenib showed a 40.3% overall response with a median response duration of 5.8 months in patients with R/R *IDH2*-mutated AML (Table 3) [4]. Ivosidenib plus azacitidine therapy for ND AML/MDS is now under evaluation in the phase 3 AGILE study [36].

Combinations with intensive chemotherapy have also been evaluated. A phase 1 clinical study revealed that ivosidenib or enasidenib in combination with standard induction chemotherapy and consolidation therapy showed 73–93% CR/CRh rates with 58–89% minimal residual disease (MRD)-negative rates in patients with *IDH1*- or *IDH2*-mutated newly diagnosed AML [37].

#### 2.2.2. Targeting Anti-Apoptotic BCL-2

**Figure 1:**
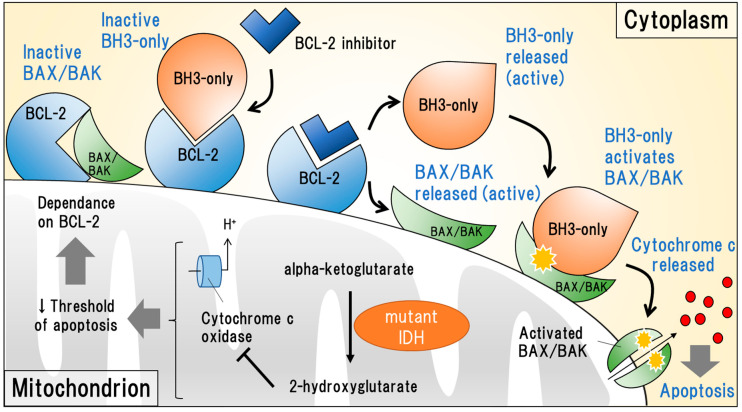
Schematic of the pharmacodynamics of B-cell leukemia/lymphoma 2 (BCL)-2 inhibitors in association with mutant isocitrate dehydrogenases (IDH)-mediated consequences. BCL-2 ordinarily inactivates key proapoptotic molecules such as BCL-2 homology 3 (BH3)-only proteins, BCL-2-associated X protein (BAX), and BCL-2 homologous antagonist/killer (BAK). BCL-2 inhibitors allow activation of these molecules to initiate the apoptotic cascade. The oncometabolite 2-hydroxyglutarate (2-HG), which is converted from alpha-ketoglutarate by mutant IDH, inhibits the activity of cytochrome c oxidase and results in decreased threshold of mitochondrial outer membrane permeabilization (MOMP), which eventually leads to dependance on BCL-2.

The B-cell leukemia/lymphoma 2 (BCL-2) family regulates the mitochondrial apoptotic pathway by controlling mitochondrial outer membrane permeabilization (MOMP) and the release of cytochrome c [38]. The family consists of proapoptotic proteins (e.g., BCL-2 homology 3 (BH3)-only, BCL-2-associated X protein (BAX), and BCL-2 homologous antagonist/killer (BAK)) and anti-apoptotic proteins (e.g., BCL-2, BCL-XL, and MCL-1) [39]. BCL-2 proteins on the mitochondrial outer membrane surface ordinarily capture cytoplasmic BH3-only proteins and inhibit BAX/BAK, which are activated by BH3-only proteins, then initiate MOMP to release cytochrome c. BCL-2 inhibitors competitively bind to BCL-2 proteins to release BH3-only proteins and disinhibit BAX/BAK, which eventually initiates the apoptotic cascade of tumor cells (Figure 1) [40]. Chan and colleagues demonstrated that the oncometabolite 2-HG inhibited the activity of cytochrome c oxidase, a key cation channel involved in the electron transport system of mitochondria, and then lowered the threshold of MOMP, resulting in dependence on BCL-2 activity (Figure 1). In this study, ex vivo and PDX model experiments showed that the BCL-2 inhibitor ABT-199 (venetoclax) significantly suppressed the proliferation of *IDH1-* or *IDH2*-mutated leukemic cells [41].

In the VIALE-A study, a phase 1b study, which proved the clinical efficacy of venetoclax in combination with azacitidine for previously untreated AML, the subgroup analysis suggested that *IDH1* and *IDH2* mutations were independently associated with a favorable prognosis (hazard ratio, 0.34) [42]. Notably, venetoclax is available for any type of AML regardless of genomic status, IDH1 and IDH2 mutations might help to predict responses to the agent. Given that FLT3 inhibition facilitates dependance on anti-apoptotic BCL-2, as mentioned above, the combination of FLT3 inhibitors and venetoclax can be a future strategy for AML [26].

### 2.3. Menin-MLL Complex-Associated Gene Mutations

#### 2.3.1. Targeting *MLL*-Rearrangement

**Figure 2:**
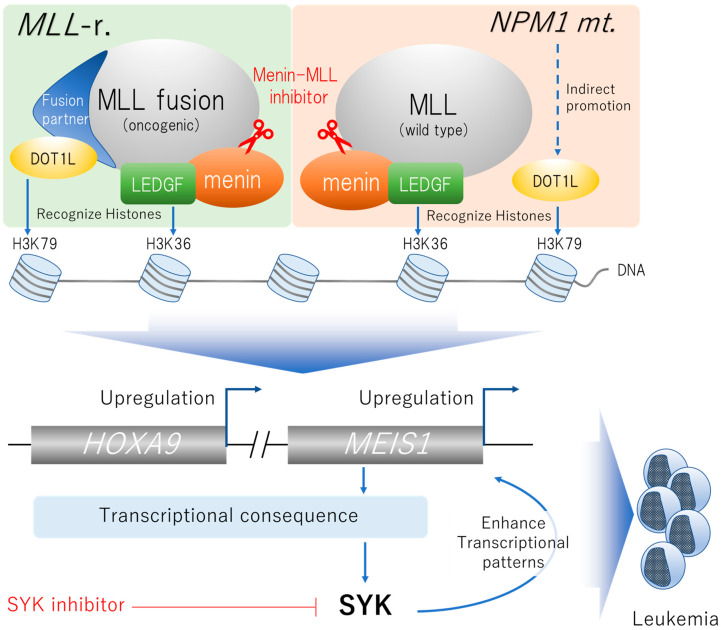
Schematic of the mechanism of menin-mediated leukemogenesis in acute myeloid leukemia (AML) with rearrangement of mixed lineage leukemia 1 (*MLL*-r.), nucleophosmin1 (*NPM1*) mutation, or nucleoporin 98 (*NUP98*) fusions. MLL fusion protein or wild-type MLL protein form chromatin-associated complex, which upregulates proliferation-initiating genes such as homeobox A9 (*HOXA9*) and myeloid ecotropic viral insertion site 1 (*MEIS1*) mediated by the histone methyltransferase telomeric silencing 1-like (DOT1L). Spleen tyrosine kinase (SYK) is involved in downstream signaling and plays a key role in *HOXA9*/*MEIS1*-overexpressing AML. Upregulated transcription of *MEIS1* results in indirect activation of SYK and, in turn, activated SYK enhances transcription of *MEIS1*.

The mixed lineage leukemia 1 (*MLL*, also known as *KMT2A*) gene encoding a histone methyl-transferase is a proto-oncogene involved in a variety of chromosomal translocations [43]. Rearrangements of the *MLL* gene (*MLL*-r.) are found in approximately 5–10% of AML, are particularly prevalent in infant leukemias, and are associated with poor prognosis and resistance to chemotherapy [1,44]. Recent studies have suggested that oncogenic MLL fusion proteins interact with the chromatin-associated complex, including disruptor of telomeric silencing 1-like (DOT1L), a histone H3K79 methyltransferase, and the product of the *MEN1* tumor suppressor gene (menin), which are required for the initiation of MLL-mediated leukemogenesis (Figure 2) [45,46]. Menin classically functions as a tumor suppressor for the endocrine lineage. However, menin also plays an essential role as a transcriptional cofactor for MLL oncoproteins [46]. Menin links MLL proteins with lens epithelium-derived growth factor (LEDGF), an epigenetic reader recognizing H3K36 histone marks, on cancer-associated target genes to upregulate the transcription [47]. In addition, oncogenic MLL fusion proteins are also linked with the histone H3K79 methyltransferase DOT1L via its fusion partners, such as AF9 [48]. Indeed, preclinical studies have suggested that leukemic cells with *MLL*-r. were dependent on DOT1L activity [49,50,51], which results in upregulation of specific genes essential for hematopoietic proliferation, such as homeobox A9 (*HOXA9*) and myeloid ecotropic viral insertion site 1 (*MEIS1*) [52,53]. Although some previous studies suggested potential therapeutic activity of DOT1L inhibitors on AML with *MLL*-r. [54,55], it turned out to be far from clinically useful [56].

Still, several preclinical studies using leukemic cell lines and xenograft mice showed on-target antileukemic effects of menin-MLL inhibitors [57,58,59,60,61]. Krivtsov and colleagues demonstrated a dramatic reduction in leukemic burden when *MLL*-r. PDX mice were treated with the orally bioavailable menin-MLL inhibitor VTP50469 [61]. This study also demonstrated uniformly suppressed expression of MLL-target genes, especially *MEIS1*, in the bone marrow of PDX mice which were treated with the menin-MLL inhibitor. Another menin-MLL inhibitor, MI-3454, also showed complete remission or regression of leukemia in a PDX model accompanied by downregulation of key leukemogenic genes such as *MEIS1* [60]. Indeed, the agent was equally effective for *MLL*-r. and *NPM1*-mutated leukemia.

#### 2.3.2. Targeting *NPM1* Mutations

Nucleophosmin1 (*NPM1*) mutations are found in approximately 30% of AML patients and often co-exist with *DNMT3A* mutations [6,62]. Although mutant *NPM1* with absent or low-allelic-ratio *FLT3*-ITD is known as a favorable prognostic factor of AML [1], *NPM1* and *FLT3* mutations are not uncommonly found simultaneously [6]. Normal NPM1 protein is a key chaperon protein in the nucleus that maintains genomic stability [63]. Mutant NPM1 protein loses the ability to be transported into the nucleus and consequently accumulates in the cytoplasm [64]. MLL proteins form a macromolecular complex involving menin that maintains expression of *HOX* genes, resulting in expansion of progenitor cells [65,66,67]. Recent studies have suggested that the interaction of menin and wild-type MLL plays a pivotal role in AML with *NPM1* mutation by upregulating leukemogenic genes, such as *HOXA*, *HOXB* and *MEIS1*, similar to the action of MLL-fusion protein (Figure 2) [62,68,69]. Aberrant demethylation of H3K79, the primary target region of DOT1L, and subsequent upregulation of *HOXA9* and *PBX3* genes have also been reported [70]. Highly potent menin-MLL inhibitors have been preclinically developed targeting *NPM1*-mutated leukemia, including MI-3454 mentioned above [60,71]. Interestingly, an in vivo study demonstrated that combined menin-MLL and FLT3 inhibition showed a synergistic anti-leukemic effect on *NPM1*-mutated and *FLT3*-mutated AML [72].

#### 2.3.3. Targeting *NUP98* Fusion

The nucleoporin 98 (*NUP98*) gene was originally identified as a component of the nuclear pore complex [73]. *NUP98* is involved in a variety of balanced translocations and inversions as the fusion partner of dozens of genes such as *HOXA9* and lysine-specific demethylase 5A (*KDM5A*), also known as *JARID1A* [74]. The majority of *NUP98* fusions are accompanied by overexpression of *HOXA9* [75,76], which is associated with poor prognosis in AML [77,78]. Recent studies have suggested that leukemic cells with *NUP98* fusions are dependent on MLL protein in terms of recruiting the fusion proteins onto the *HOXA* locus to initiate leukemogenesis [79]. Given the synergy of *MLL*-r. and *NPM1*-mutated leukemia, Heikamp and colleagues demonstrated that the menin-MLL inhibitor VTP50469 prolonged survival of PDX mice with human leukemia following implantation with *NUP98*-*HOXA9* and *NUP98*-*JARID1A*, along with suppressed pro-leukemic gene expression and upregulated differentiation markers [80].

#### 2.3.4. Targeting SYK Signaling

Spleen tyrosine kinase (SYK) was originally identified as a signaling molecule downstream of the B cell antigen receptor. SYK also plays a key role in AML in terms of phosphorylating signal transducer and activator of transcription 5 (STAT5) [81] and cooperating with *FLT3*-ITD in maintaining leukemia [82], and is also associated with an unfavorable prognosis [83]. Mohr and colleagues revealed that Meis1 induced SYK signaling through multiple transcriptional events, including downregulation of microRNA(miR)-146a, which negatively regulates SYK expression in Hoxa9-overexpressing myeloid progenitor cells. The study also showed that SYK overexpression enhanced Meis1 transcriptional patterns, resulting in dependence on SYK activity in *Hoxa9*/*Meis1*-overexpressing myeloid progenitors. Indeed, SYK inhibition prolonged survival of mice with Hoxa9/Meis1-driven leukemia [84]. In an international multicenter phase 1b/2 study, 34 previously untreated AML patients were treated with the SYK inhibitor entospletinib in combination with standard induction chemotherapy, resulting in a 56% CR rate with acceptable toxicity [85]. In this study, patients with *HOXA9*/*MEIS1* overexpression showed significantly better OS (HR 0.32, 95% CI 0.100–0.997) than others (Table 4)

### 2.4. TP53 Mutations

#### 2.4.1. p53 Stabilizers

Tumor protein p53 (TP53) is a major tumor suppressor inducing growth arrest or apoptosis. TP53-induced apoptosis is partially mediated by stimulation of BAX and repression of BCL-2 expression [86,87]. TP53 prevents activity of cyclin-dependent kinase (CDK) 7, a part of CDK-activating kinase (CAK), to stop cell cycle in response to DNA damage [88]. TP53 gene mutations are found in less than 10% of AML patients, is one of the most well-recognized adverse genomic factors, and is often associated with complex karyotype [1,6]. A number of in vitro and in vivo studies revealed that TP53-mutated leukemia gains enhanced self-renewal capacity and a competitive growth advantage, subsequently accumulating additional mutations such as *DNMT3A*, *TET2*, and *ASXL1* [89]. APR-246 (eprenetapopt) is the first-in-class anti-tumor agent targeting *TP53* mutation, which thermodynamically stabilizes p53 protein and shifts the equilibrium toward a functional conformation [90]. In a phase 1b/2 clinical study, the combination therapy of APR-246 and azacitidine produced a 71% overall response rate, including a 41% CR rate, in patients with *TP53*-mutated high-risk MDS or AML (Table 4) [91]. Despite this promising result, the combination therapy did not meet the primary endpoint (CR rate) in a phase 3 clinical trial for patients with *TP53*-mutated MDS, though the CR rate tended to be superior in the combination group than the azacitidine monotherapy group (33.3% vs. 22.4%) [92]. The next-generation p53 stabilizer APR-548 is now being evaluated in an early phase clinical trial [93].

#### 2.4.2. Targeting CD47

The transmembrane protein CD47, also known as the “don’t-eat-me signal”, is the ligand for signal regulatory protein alpha (SIRPα) on macrophages and dendritic cells and results in inhibition of phagocytosis [94]. Increased expression of CD47 on AML stem cells has been shown to be associated with poor prognosis [95]. The anti-CD47 antibody magrolimab in combination with azacitidine showed a 57% CR/CRh rate in patients with treatment-naïve AML (65% had *TP53* mutation) who were ineligible for intensive therapy in a phase 1b study. Although the immune escape mechanism through CD47 would not be specific for *TP53*-mutant AML, the CR/CRh rates were somewhat higher (67%) in *TP53*-mutated AML in this study (Table 4) [96].

### 2.5. Other Potential Molecular-Targeting Agents Currently Available in Solid Tumors

#### 2.5.1. KIT Inhibitors

KIT protein, also known as CD117, is expressed in 70% of AML as well as normal hematopoietic progenitor cells. KIT mutations are seen in less than 10% of all subsets of AML and in approximately 30% of the core-binding factor (CBF) AML [97,98,99]. *KIT* mutations are associated with worse prognosis in CBF-AML [2,100]. KIT protein is a cell-surface receptor for the cytokine SCF (Kit ligand) and plays an essential role in anti-apoptosis, proliferation, and hematopoiesis via the Ras-Erk pathway, the PI3K/AKT pathway, and the JAK/STAT pathway [101,102]. Activating mutations of *KIT* gene, commonly on the exon 8 and 17, are frequently found in gastrointestinal stromal tumor (GIST) and systemic mastocytosis, as well as CBF-AML. A number of tyrosine kinase inhibitors (TKIs) targeting KIT such as imatinib, sunitinib sorafenib, regorafenib, dasatinib, nilotinib, ponatinib, and midostaurin are suggested to be a potential therapeutic agent for *KIT*-mutated tumors [103]. A part or all of imatinib, sunitinib, regorafenib and avapritinib are involved in the standard treatment for GIST and systemic mastocytosis. While some *KIT* A-loop mutations (e.g., D816 alterations) render resistance to imatinib and sunitinib in patients with GIST, some agents (e.g., avapritinib and ponatinib) remain effective for such resistant cases [104,105]. Although the clinical role of KIT inhibitors in treatment of AML has not been established, there are a few clinical reports suggesting an anti-leukemic effect of KIT inhibitor (e.g., dasatinib) for *KIT*-mutated AML [106,107].

Kampa-Schittenhelm and colleagues reported 77-years-old patient with *KIT* D816V-mutated CBF-AML which relapsed after the first-line decitabine monotherapy [106]. Upon careful consideration and informed consent, he received a KIT inhibitor dasatinib as salvage chemotherapy. Peripheral blasts started to reduce on day 15 and, instead, neutrophils/monocytes increased in a few days, suggesting the release of differentiation blockade. The patient’s mononuclear cell sample showed dose-dependent cytoreduction ex vivo responding to dasatinib and vanishment of phosphorylated KIT proteins on Western immunoblotting after dasatinib administered. Although the patient eventually shifted to best supportive care because of inacceptable tolerability of dasatinib, this report provided proof-of-concept that KIT inhibition has anti-leukemic effect on *KIT*-mutated CBF-AML.

Recently, a heat shock protein (HSP)-90 inhibitor pimitespib (TAS-116) showed significant improvement of PFS in patients with GIST resistant to imatinib, sunitinib, and regorafenib in the phase 3 CHAPTER-GIST-301 trial [108]. HSP-90 is a molecular chaperone that engages a variety of clients including KIT by interacting with co-chaperone proteins [109]. Thus, HSP-90 inhibition results in reduced functional stability of KIT then attenuates it’s signaling. Preclinical studies have suggested that inhibition of HPS-90 led to tumor shrinkage in human tumor xenograft mouse model as well as depletion of multiple HSP-90 clients [110]. Katayama and colleagues reported that HSP-90 inhibition restored the sensitivity to FLT3 inhibitors in AML cell-lines with *FLT3*-ITD plus *FLT3*-TKD (e.g., N676K, F691L, D835V, and Y824C) which render resistance to FLT3 inhibition [111]. HSP90 inhibitors in combination with other TKIs can be a future strategy in AML treatment.

#### 2.5.2. Targeting RAS Pathway-Related Genes

RAS proteins have GTPase activity responding to growth factor receptor activation and play an essential role in cell proliferation [112,113]. *RAS* genes (e.g., *KRAS*, *NRAS*, and *HRAS*) are one of most popular proto-oncogenes and frequently mutated in human cancer, affecting the mitogen-activated protein kinase (MAPK), phosphatidylinositol-3-kinase (PI3K), and Ras-like (Ral) small GTPase (RalGEF) signaling pathways. Activating mutations of *NRAS* and *KRAS* are found in approximately 15–25% of patients with AML [6]. *RAS* genes commonly co-mutated with RAS-regulating genes (e.g., *PTPN11* and *NF1*) and/or signaling receptor genes that rely on RAS-involving pathways (e.g., FLT3 and KIT) [6,114]. Recent studies of cancer clonal evolution have suggested that additional RAS mutations are associated with resistance to FLT3 inhibitors in *FLT3*-mutated AML. McMahon and colleagues sequentially analyzed clinical samples from patients with *FLT3*-mutated AML which progressed on gilteritinib treatment [115]. Among forty-one participants of this study, fourteen patients (34%) acquired new *NRAS* and/or *KRAS* mutations after progression on FLT3 inhibition. Almost complete substation of *RAS*-mutated subclones for the original *RAS*-wild *FLT3*-mutated clone was observed in a few cases. In addition, leukemic cell lines with both *FLT3* and *RAS* mutations showed resistance to gilteritinib monotherapy, which was canceled by co-administration of a MEK inhibitor trametinib.

Preclinical experiments have shown that inhibition of both MAPK and PI3K pathway did not cause significant leukemic cell death but led to static effects instead [116,117]. Similarly, early phase clinical trials evaluating MEK or AKT inhibitor in patients with relapsed/refractory AML demonstrated only transient or little anti-leukemic effects [118,119]. Whereas, Pomeroy and colleagues found that *NRAS*-driven AML cell line relapsed after genetic suppression on *NRAS* (NRI-AML) was devoid of MAPK and PI3K signaling but dependent on the cyclin-dependent kinase (CDK) 5-mediated activation of RalGEF signaling. In in vivo experiments using PDX mouse model, a CDK inhibitor dinaciclib which inhibits CDK5-mediated RalGEF signaling induced leukemic cell apoptosis and prevented evolution of NRI-AML, suggesting that RalGEF signaling is involved in escaping mechanism of *RAS*-mutated AML and can be a potential therapeutic target.

The KRAS^G12C^-specific inhibitor sotorasib demonstrated 7–32% of overall response rates in advanced solid tumors (mainly non-small cell lung cancer and colorectal cancer) with *KRAS* G12C mutation in a phase 1 study [120] and now available in some countries. The efficacy of *KRAS* inhibition have not been proved in hematologic malignancies neither clinically nor preclinically. However, given that activating *RAS* mutations are associated with worse prognosis and drug resistance in AML, KRAS inhibition might be included in future possibility.

## 3. Conclusions and Perspective

Molecular-targeted therapies, especially for specific genomic abnormalities, have had significant impacts on AML treatment. FLT3 inhibitors and IDH inhibitors have proved its efficacy in clinical trials and now available in practice. In addition, menin-MLL inhibitors have shown anti-leukemic effects in preclinical studies of AML with menin-related genomic alterations such as *MLL* rearrangements, *NPM1* mutations, and *NUP98* fusion genes. SYK inhibitors can be another strategy for AML with menin-related genomic alterations. For AML with *TP53* mutation, p53 stabilizers and anti-CD47 antibodies can be a candidate for gene-specific therapeutic agents. Although clinical evidence has not been sufficient yet, anti-KIT strategy such as HSP-90 inhibitors and RAS-pathway interference might be a future approach for AML treatment. Mutations of *FLT3*, *MLL*, and *TP53* are considered to be unfavorable prognostic factors so far. However, the development of targeted therapies with or without conventional therapy may improve or perhaps reverse the current situation. Aberrant oncogenic mechanisms depending on specific genomic abnormalities can be an ideal therapeutic target, though preventing or overcoming treatment resistance remains a challenge. Determining genomic features and following case-specific molecular-targeted strategy should be future therapeutic strategy in AML. Results from current developments and ongoing trials are anticipated.

## Figures and Tables

**Table 1:** The phase 3 trials of currently available FLT3 inhibitors.

Author and Jounal	Object(s)	Disease State	Agent(s)	Phase	Response Rate		Median Survival
Stone, et al. [14] N Engl J Med 2017	*FLT3*-mutated AML (both ITD and TKD)	ND	Midostaurin	III	CR	70% (504/717)	8.2 mo. [5.4–10.7]
			+ StdCTx				
Perl, et al. [15] N Engl J Med 2019	*FLT3*-mutated AML (both ITD and TKD)	R/R	Gilteritinib	III	CR/CRi PR	54% (134/247) 13% (33/247)	9.3 mo. [7.7–10.7]
Cortes, et al. [16] Blood 2019	*FLT3*-mutated AML (ITD only)	R/R	Quizartinib	III	CR/CRi	48% (118/245)	18.5 mo. [10.8–28.8]

StdCTx: standard chemotherapy, CRi: CR with incomplete hematologic recovery, PR: partial response. ITD: internal tandem duplication, TKD: mutation of tyrosine kinase domain. ND: newly-diagnosed, R/R: relapsed or refractory to previous therapy.

**Table 2:** Clinical studies evaluating FLT3 inhibitors in combination with low-intensity chemotherapy.

Author and Jounal	Object(s)	Disease State	Agent(s)	Phase	Response Rate		Median Survival
Ohanian, et al. [22] Am J Hematol 2018	*FLT3*-mutated AML (ITD only)	NDi	Sorafenib + AZA	I/II	CR	26% (7/27)	7.1 mo. [1,2,3,4,5,6,7,8,9,10,11,12,13,14,15,16,17,18,19,20,21,22,23,24,25,26,27,28,29]
Strati, et al. [23] Am J Hematol 2015	*FLT3*-mutated AML or MDS (both ITD and TKD)	NDi or R/R	Midostaurin + AZA	I/II	ORR	26% (14/48)	22 wk. [15,16,17,18,19,20,21,22,23,24,25,26,27,28,29]
Swaminathan, et al. [24] Haematologica 2020	*FLT3*-mutated AML or MDS (ITD only)	NDi or R/R	Quizartinib + AZA/LDAC	I/II	CR CRi	17% (12/70) 37% (26/70)	19.2 mo. for AZA 8.5 mo. for LDAC
Wang, et al. [25] Blood 2020	*FLT3*-mutated AML (both ITD and TKD)	NDi	Gilteritinib + AZA	III	Did NOT meet the primary endpoint 39% were alive when study halted		

NDi: newly-diagnosed and ineligible to intensive chemotherapy, AZA: azacitidine, LDAC: low-dose cytarabine.

**Table 3:** Clinical studies evaluating IDH inhibitors with or without cytotoxic agents.

Author and Jounal	Target(s)	Disease State	Agent(s)	Phase	Response Rate		Duration of Response
DiNardo, et al. [3] N Engl J Med 2018	*IDH1*-mutated AML	R/R	Ivosidenib	Ib	CR ORR	21.6% (56/258) 41.6% (107/258)	8.2 mo. [5.5–12.0]
Stein, et al. [4] Blood 2018	*IDH2*-mutated AML	R/R	Enasidenib	Ib/II	CR ORR	19.3% (34/176) 40.3% (71/176)	5.8 mo. [3.9–7.4]
DiNardo, et al. [36] N Engl J Med 2018	*IDH1*-mutated AML	NDi	Ivosidenib + AZA	Ib	CR ORR	60.9% (14/23) 78.3% (18/23)	Not reached Median f/u 16 mo.
Stein, et al. [37] Blood 2018	*IDH1*-mutated AML	ND	Ivosidenib + StdCTx	I	ORR	78.0% (32/41)	41% proceeded to HSCT
	*IDH2*-mutated AML		Enasidenib + StdCTx		ORR	68.8% (53/77)	43% proceeded to HSCT

AZA: azacitidine, StdCTx: standard chemotherapy, HSCT: hematopoietic stem cell transplantation, f/u: follow-up.

**Table 4:** Clinical trials evaluating SYK inhibitor, p53 stabilizer, and anti-CD47 antibody.

Author and Reference	Category	Object(s)	Disease State	Agent(s)	Phase	Response Rate		Median Survival
Walker, et al. [85] Clin Cancer Res 2020	SYK inhibitor	de novo AML	ND	Entospletinib + StdCTx	Ib/II	CR CR/CRi	56% (19/34) 71% (24/34)	37.1 mo. [16.8–Inf.]
Sallman, et al. [91] J Clin Oncol 2021	p53 stabilizer	*TP53*-mutated AML or MDS	HMA-naïve	Eprenetapopt + AZA	Ib/II	ORR *	64% (7/11)	10.8 mo. [8.1–13.4]
NCT03745716 [92]	p53 stabilizer	*TP53*-mutated MDS	HMA-naïve	Eprenetapopt + AZA (vs. AZA alone)	III	Did NOT primary endpoint (CR 33.3% vs. 22.4%)		
Sallman, et al. [96] ASH meeting 2020	Anti-CD47 antibody	AML	NDi	Magrolimab + AZA	Ib	CR ORR	44% (15/34) 65% (22/34)	12.9 mo. **

StdCTx: standard chemotherapy, ND: newly-diagnosed, NDi: newly-diagnosed and ineligible to intensive chemotherapy. * only for AML. ** *TP53* mt.

## Data Availability

Data sharing not applicable.
